# 22q11.2 Deletion Syndrome in Taiwan: Clinical Presentation and Immune System Status of Patients

**DOI:** 10.7150/ijms.86773

**Published:** 2023-09-04

**Authors:** Chung-Lin Lee, Shan-Miao Lin, Ming-Ren Chen, Chih-Kuang Chuang, Huei-Ching Chiu, Yuan-Rong Tu, Yun-Ting Lo, Ya-Hui Chang, Hsiang-Yu Lin, Shuan-Pei Lin

**Affiliations:** 1 Department of Pediatrics, MacKay Memorial Hospital, Taipei, Taiwan.; 2 Institute of Clinical Medicine, National Yang-Ming Chiao-Tung University, Taipei, Taiwan.; 3 Department of Rare Disease Center, MacKay Memorial Hospital, Taipei, Taiwan.; 4 Department of Medicine, Mackay Medical College, New Taipei City, Taiwan.; 5 Mackay Junior College of Medicine, Nursing, and Management, Taipei, Taiwan.; 6 Division of Genetics and Metabolism, Department of Medical Research, MacKay Memorial Hospital, Taipei, Taiwan.; 7 College of Medicine, Fu-Jen Catholic University, Taipei, Taiwan.; 8 Department of Medical Research, China Medical University Hospital, China Medical University, Taichung, Taiwan.; 9 Department of Infant and Child Care, National Taipei University of Nursing and Health Sciences, Taipei, Taiwan.; Chung-Lin Lee and Shan-Miao Lin contributed equally to this study.

**Keywords:** 22q11.2 deletion syndrome, congenital heart disease, intellectual disability, T-cell defect, Taiwan

## Abstract

**Background:** 22q11.2 deletion syndrome (22q11.2DS) is a microdeletion syndrome exhibiting significant clinical phenotype variability. This study aimed to investigate the clinical features, immune profiles, and cognitive abilities of 22q11.2DS patients receiving treatment at MacKay Memorial Hospital in Taipei, Taiwan.

**Methods:** This is a cross-sectional analysis between January 2001 and December 2022. We recruited 27 patients with 22q11.2DS using fluorescence *in situ* hybridization (FISH), multiplex ligation-dependent probe amplification (MLPA) and array comparative genomic hybridization (aCGH). Our evaluation included patient history, physical examination, laboratory analysis, and cardiac and cognitive assessment.

**Results:** We included 27 patients with 22q11.2DS, 7 (25.9%) of whom were female. The median age of the patients was 17.9 yr. Ninety-three percent of the patients exhibited the characteristic facial features associated with the syndrome. A family history of 22q11.2DS was found in 11.1% of the patients. Furthermore, 74.1% of the patients had a congenital heart defect, the most common of which was tetralogy of Fallot (40.7%). Hypocalcemia was observed in 40.7% of the patients. A low T-cell count was observed in 66.7% of the patients, whereas 18.5% had low immunoglobulin levels. Cognitive assessments revealed that four out of six evaluated patients (66.7%) had an intellectual disability, as evidenced by intellectual quotient scores less than 70. The remaining two patients (33.3%) had a borderline intellectual function.

**Conclusion:** Tetralogy of Fallot, hypocalcemia, immunologic defects, and cognitive impairment were common among our patients. To address the potential multisystem involvement, we recommend that all affected individuals undergo a comprehensive evaluation by a multidisciplinary care team.

## Introduction

22q11.2 deletion syndrome (22q11.2DS) is the most common microdeletion syndrome in humans 1. It is caused by deletions on the long arm of chromosome 22 2. 22q11.2DS was previously known as a velocardiofacial syndrome, conotruncal anomaly face syndrome, Cayler cardiofacial syndrome, and, in some cases, autosomal dominant Opitz G/BBB syndrome 3,4. Fluorescence *in situ* hybridization (FISH), multiplex ligation-dependent probe amplification (MLPA) and array comparative genomic hybridization (aCGH) are often used diagnostic test for 22q11.2DS 2,5.

Two separate studies have reported that the estimated prevalence of 22q11.2DS ranges from 1 in 4,000 to 1 in 7,092 live births 1,6. Around 90% of cases were caused by de novo mutations, whereas the remaining cases were caused by inherited chromosome 22 with deletion at the q11.2 arm from one parent in an autosomal dominant pattern 2,7,8. Patients with 22q11.2DS have a wide range of clinical manifestations, including a conotruncal congenital heart defect, immunodeficiency, characteristic facial features, palatal defects, developmental and/or learning disabilities, hypocalcemia, hypoparathyroidism, and hypothyroidism. Additional abnormalities linked to 22q11.2DS include renal anomalies, hearing loss, growth retardation, psychiatric disorders, and feeding and swallowing difficulties. Proposed practical guidelines for managing patients with 22q11.2DS suggest strategies for identifying, assessing, monitoring, and treating associated morbidities 3,9. These guidelines recommend that all individuals affected by the condition undergo regular comprehensive evaluations 9.

Due to the lack of comprehensive, integrated studies on 22q11.2DS in Taiwan, this study aimed to outline the clinical characteristics, immunological features, and intellectual status of patients with 22q11.2DS at MacKay Memorial Hospital to facilitate early identification and intervention of the condition in an effort to reduce morbidity and mortality.

## Materials and Methods

The medical records of patients at MacKay Memorial Hospital from 2001 to 2022 were analyzed using a hospital-based, cross-sectional design. The study identified 27 patients with 22q11.2DS, which were confirmed by FISH, MLPA or aCGH analysis. Between January 2001 and December 2022, electronic medical records of patients were reviewed, as well as clinical history interviews and physical examinations to evaluate growth and development, family history, facial malformation, congenital heart diseases, history of hypocalcemia, urological imaging, feeding difficulties, infections, and vaccinations. All 27 participants were also invited to undergo further evaluation of their intellectual status and laboratory tests.

### Genetic testing

FISH analysis was performed using Vysys LSI TUPLE1 SpectrumOrange/LSI ARSA SpectrumGreen probes, which bind to band 22q11.2 loci D22s553, D22S609, and D22S942 (LSI DiGeorge/VCFS, TUPLE1-HIRA locus SpectrumOrange, Abbott®), as well as band 22q13 (LSI ASRA SpectrumGreen, Abbott®), to identify a deletion of chromosome 22q11.2. At least 20 metaphases were analyzed in each FISH assay. We utilized the MLPA P034/P035 kit (MRC, Holland). The DNA amplification products were then analyzed through capillary electrophoresis using the ABI 3500Dx Genetic Analyzer (Applied Biosystems Life Technologies, Singapore). Subsequently, the obtained results were analyzed with the MLPA Cofalyser.net software (MRC, Holland). For aCGH, we employed the Affymetrix GeneChip Genome-Wide Human SNP array 6.0 (Affymetrix, Santa Clara, CA, USA). This array comprises 750,000 probes with a resolution of 200 kb throughout the genome to detect copy number variation. The array data were analyzed using Affymetrix Genotyping Console™ version 3.0.1, following the instructions provided by the manufacturers to handle all samples.

### Intellectual testing

A developmental-behavioral pediatrician and a licensed clinical psychologist evaluated the intellectual status of the patients, and the patients were divided into four age-based categories for assessment using four different tools to evaluate intellectual quotient (IQ): the Bayley Scales of Infant and Toddler Development third edition (Bayley-III), the Stanford-Binet Intelligence scales, the Wechsler Intelligence Scale for Children (WISC)-fourth edition, and the Wechsler Adult Intelligence Scale (WAIS)-fourth edition. These tools were administered to patients aged birth to 3, 3-7, 7-17, and >17 yr, respectively. A psychiatric diagnosis was also recorded. An IQ score of less than 70 (2 standard deviation below the mean) was considered intellectually disabled, whereas an IQ score of 70-84 was classified as borderline intelligence (1 to 2 *SD* below the mean) 10.

### Laboratory testing

The laboratory tests obtained included the complete blood count, as well as measurements of various lymphocyte subpopulations, such as CD3+, CD4+, and CD8+, levels of immunoglobulins (IgG, IgA, IgM, and IgE), serum calcium and phosphate levels, thyroid hormone measurements (thyroid-stimulating hormone and free T4), as well as parathyroid hormone levels. After comparing them to the normal 95% confidence limits for the individual's age, low total lymphocyte and immunoglobulin levels were defined as lymphopenia and low immunoglobulin levels. The absolute counts and percentages of CD3+, CD4+, and CD8+ were also found to be low, with values below the 10th percentile based for age 11,12. Hypocalcemia (corrected for serum albumin level), hypophosphatemia, hypothyroidism, and hypoparathyroidism were below the 10th percentile of the age distribution 13.

### Statistical analysis

MedCalc version 20.218 (MedCalc Software Ltd, Ostend, Belgium) was used for statistical analysis. The Wilcoxon signed‐rank test was used for small, numbered groups. A descriptive analysis was used to characterize the study population. Continuous variables were expressed as the median, whereas categorical variables were reported as a percentage or ratio, as deemed appropriate. The statistical data were presented as mean ± standard deviation, with a significance level of *p* < 0.05. A *p* value <0.05 was statistically significant.

## Results

Based on genetic testing, the study included 27 patients with 22q11.2DS, with males accounting for 74.1% (20 patients). The clinical characteristics of these patients are shown in **Table 1**. At the time of the study, the median age of the patients was 17.9 yr (interquartile range [IQR], 14.1-20.3). The majority of patients had clinical symptoms during their newborn period, and the median age at diagnosis was 0.8 yr (IQR, 0.3-7.2). Depending on specific symptoms, the age of onset, age at diagnosis, and diagnostic criteria differed significantly. Furthermore, 11.1% of patients had a confirmed family history of the deletion, including two siblings.

Typical facial malformations, such as palpebral fissure narrowing, hypertelorism, widened nasal dorsum, hypoplastic nasal alae, nasal voice, short philtrum, micrognathia, and malar flattening, were found in 25 patients (92.6%). Seven patients (25.9%) had submucosal cleft palate, whereas one patient (3.7%) had a cleft lip. Only one patient (3.7%) had both cleft lip and submucosal cleft palate. Congenital heart disease was the most common presenting symptom, and most patients were referred to MacKay Memorial Hospital for cardiac evaluation and treatment.

Congenital cardiovascular anomalies were diagnosed in 20 patients (74.1%), with details of the findings presented in** Table 2**. The most common cardiac defect was tetralogy of Fallot (TOF), found in 11 patients (40.7%), followed by ventricular septal defect (VSD, 40.7%), atrial septal defect (14.8%), and patent ductus arteriosus (11.1%). These primary anomalies were frequently associated with subpulmonic VSD (25.9%), right-sided aortic arch (22.2%), major aortopulmonary collateral arteries (14.8%), aberrant subclavian artery (11.1%), and double aortic arch (11.1%).

After reviewing the electronic medical records, it was found that 11 patients (40.7%) had a history of hypocalcemia. All four patients who underwent hypoparathyroid hormone level testing (14.8%) had a history of hypocalcemia. Additionally, one patient (3.7%) had primary hypothyroidism, and three (11.1%) had primary hyperthyroidism.

The immunological evaluation was conducted for all our patients. Five patients (18.5%) had low serum immunoglobulin levels, including two with pan hypogammaglobulinemia, one with low IgA and IgG levels, and one with low IgA and IgE levels. Lymphopenia was observed in 29.6% of patients compared with age-specific normal values. T-cell deficiency were found in 11 patients (40.7%), as determined by lymphocyte-subset enumerations using flow cytometry. Of these patients, seven (25.9%) had low absolute CD3+ counts or percentages, one (3.7%) had low CD4+ counts or percentages, and three (11.1%) had low CD8+ counts or percentages. None of the patients required intravenous immunoglobulin replacement therapy. There was no evidence of an autoimmune disease. The laboratory profiles for both immunologic and hormonal parameters can be found in **Table 3**. The thymus was evaluated in 11 patients using a computed tomography scan or a surgical procedure. Four of the 11 (36.4%) assessed were determined to have a nonvisualized or hypoplastic thymus.

**Figure 1:**
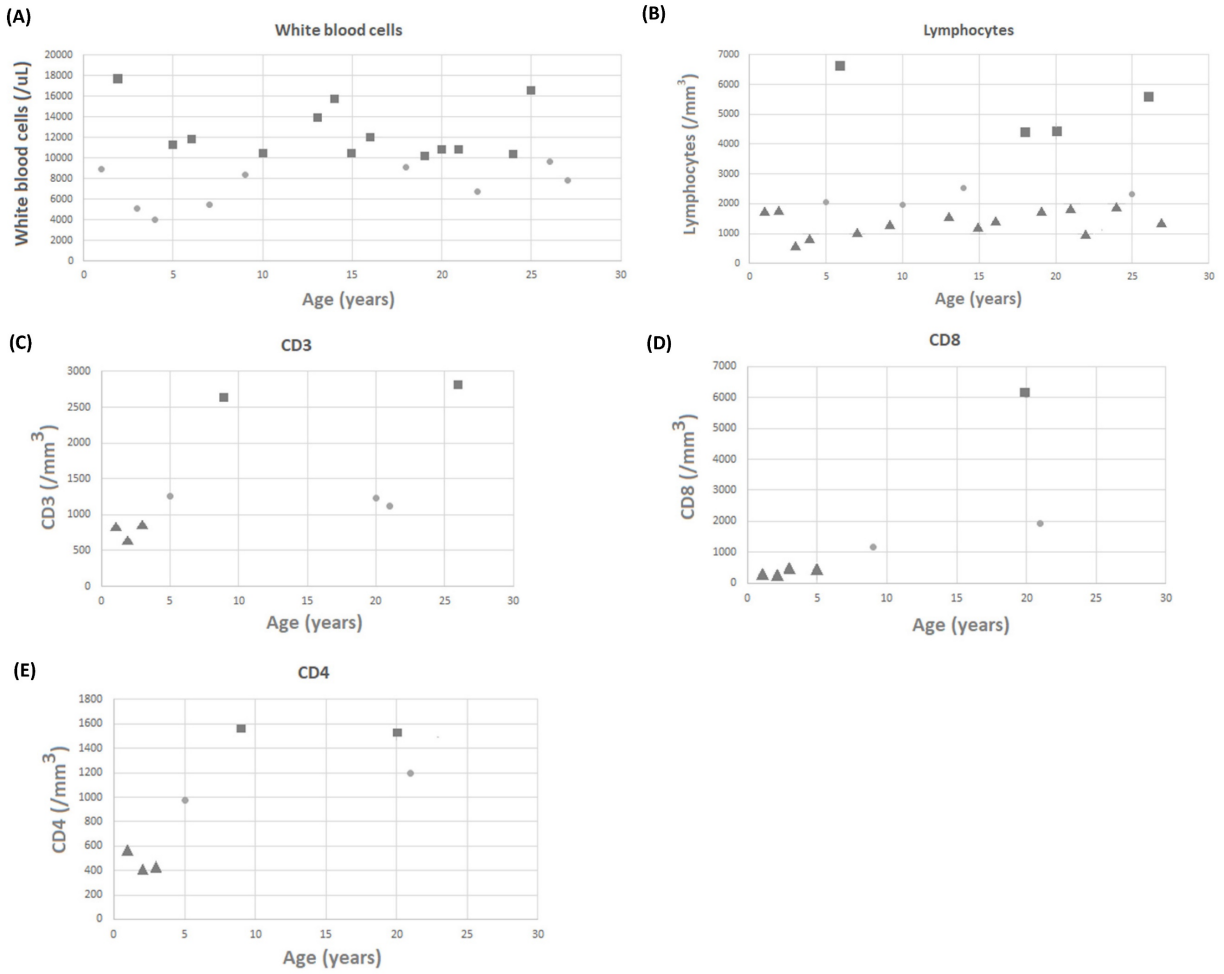
The scattergram of age and (A) white blood cells (WBCs), (B) lymphocytes, (C) CD3 cells, (D) CD8 cells, and (E) CD4 cells. Circle symbols represent biomarker concentrations within the normal range, square symbols represent concentrations above the upper limit of normal, and triangle symbols represent concentrations below the lower limit of normal.

**Figure 2:**
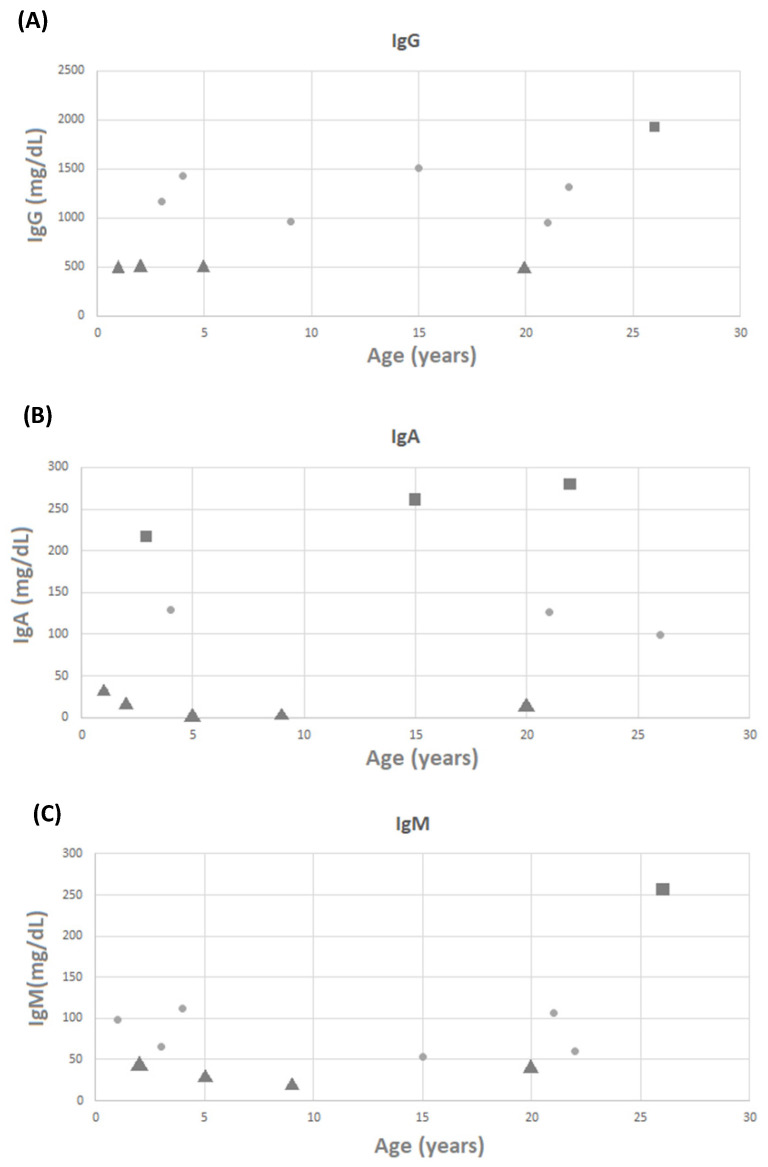
The scattergram of age and (A) IgG, (B) IgA, and (C) IgM. Circle symbols represent biomarker concentrations within the normal range, square symbols represent concentrations above the upper limit of normal, and triangle symbols represent concentrations below the lower limit of normal.

White blood cell counts for 22 patients were recorded, with a mean value of 10.32 ± 3.51 k/mL **(Figure 1A)**. Lymphocyte counts for 22 patients were recorded, with a mean value of 3,010.87 ± 1,708.24 lymphocytes/mm^3^ **(Figure 1B)**. There were no patients with low white blood cell counts; however, 18 of 22 patients had low lymphocyte counts. Three (11.1%) and four (14.8%) of the 27 patients with available data on CD3 and CD8 levels had low levels, respectively **(Figure 1C and 1D)**. Three (11.1%) of the 27 patients with available data on CD4 levels had low levels **(Figure 1E)**. It is worth noting that none of the patients required any specific treatment for these laboratory abnormalities. IgG, IgM, and IgA levels were measured in 27 patients, with mean values of 1,024.45 ± 498.22, 80.27 ± 66.28, and 107.36 ± 105.46 mg/dL, respectively. Four patients had low IgG levels, five had low IgA levels, and four had low IgM levels **(Figure 2A-2C)**.

The formal intellectual analyses were conducted on six patients, representing 22.2% of the total sample **(Table 4)**. The median age at evaluation was 5.6 yr, with an interquartile range (IQR) of 3.4-8.1. The median full-scale IQ (FSIQ) of all evaluated patients was 67, ranging from 54 to 83. The Bayley-III scale was used for patients under 3 years old, and the Stanford-Binet Intelligence scales were used for those aged 3-7 years old. WISC-IV was used to evaluate patients aged 7-17 yr, and WAIS-IV was used to assess patients older than 17 yr. According to the previously mentioned definitions, four (66.7%) of the six patients were diagnosed with intellectual disability, and the remaining two (33.3%) were classified as having borderline intelligence (FSIQ, 70-84). In terms of the factors that can affect the IQ score, no statistical difference was found between the group with major cardiovascular diseases and the group with minor cardiovascular diseases. The median FSIQ score for patients with major cardiovascular diseases was 66.0 (IQR, 63.5-71.0) and 68.0 (IQR, 61.0-75.5) for those with minor cardiovascular diseases.

## Discussion

This report presents the clinical characteristics of patients with 22q11.2DS in Taiwan, with no discernable gender bias. Although the onset of symptoms occurred during the newborn period, the genetic diagnosis was not confirmed until the average age of 18 months. Most patients were referred to MacKay Memorial Hospital for congenital heart disease, with TOF being the most common cardiac condition. All patients who were evaluated intellectually had developmental delays or intellectual impairments. These findings confirm that 22q11.2DS affects multiple systems and has a diverse range of phenotypic expressions.

22q11.2DS is a disorder caused by haploinsufficiency, which means that an affected parent has a 50% probability of passing the condition on to their offspring 7. Nonetheless, most cases of 22q11.2DS are caused by spontaneous or de novo mutations. Peer-reviewed studies indicate that only 10% of cases are inherited from affected parents 1-3, which is consistent with our finding that 11.1% of patients had a history of parents with confirmed chromosomal deletions. Because not all families of the participants were evaluated for 22q11.2 deletions, the reported proportions of inherited chromosomal deletions may be underestimated. In addition, predicting the phenotype in a sibling inheriting the 22q11.2 deletion is unreliable due to the substantial intrafamilial clinical variability linked to 22q11.2 deletion syndrome 14. In the three cases of patients with affected parents, the patients exhibited the characteristic facial phenotypes of 22q11.2DS, similar to their affected parents. However, distinct variations were observed between generations, with one patient presenting with a cleft palate and another experiencing seizure, neither of which were observed in the parents.

The gold standard technique for detecting this microdeletion is the SNP microarray with a probe targeting the 22q11.2 chromosome region 5,14. This method is widely used and can identify the proximal and common deletion in more than 90% of patients with 22q11.2DS 5,16. Despite its high sensitivity, a negative FISH result does not completely rule out the possibility of 22q11.2DS, as some patients may have minor or atypical deletions not detected by this technique. In such cases, chromosomal microarray analysis can be a valuable tool for diagnosing clinically suspected patients who have tested negative for FISH 1,5. The 22q11.2DS phenotype is believed to be primarily influenced by several genes. *TBX1*, *HIRA*, *UFD1L*, and *CRKL* are some genes associated with developing cardiac and palatal anomalies 16. We were unable to pinpoint the exact genes involved because we used the FISH technique, which resulted in the missing identification of patients with atypical deletions.

According to a previous study, the most common cardiac malformations associated with 22q11.2DS include truncus arteriosus, TOF, and interrupted aortic arch, all of which fall under the category of conotruncal heart defects 17. In this study, the proportion of TOF among conotruncal heart defects was found to be 72%, which is notably higher than the percentages reported in Western countries (ranging from 13% to 43%) 17, 18. This finding implies a higher incidence of TOF in Asian patients with 22q11.2DS than in Caucasian patients 15.

22q11.2DS has a wide range of clinical manifestations, and its heterogeneity of causes often leads to confusion 3, 18. Hypocalcemia due to hypoparathyroidism is a common presentation that can develop at any age but is more common after puberty 9. In this study, 40.7% of the patients had a history of hypocalcemia, which is consistent with a large study of adults 19 but higher than most previous studies 3, 9. Four percent of the patients showed abnormal serum thyroid hormone levels. Previous studies have found hypothyroidism and hyperthyroidism in patients with 22q11.2DS 3, 9, although the underlying mechanisms are unclear, and it is unknown whether they are caused by autoimmune or developmental factors 1.

The prevalence of T-cell and T-cell subset deficiencies in our cohort was high, with a notably higher incidence of CD4+ deficiency before the age of 2 yr. These findings are consistent with existing literature demonstrating various T-cell deficiencies in patients with 22q11.2DS due to thymic hypoplasia 20, 21, which is primarily prevalent in the pediatric age group 22, 23. A study of 34 individuals with 22q11.2DS reported that CD4+ T-cell levels normalized in most individuals up to the age of 3 yr 24. In contrast to previous studies reporting higher rates of IgM deficiency in patients with 22q11.2DS 25-27, our study found that the most prevalent humoral deficiency was IgA deficiency.

Immunodeficiency develops due to malformation of the third and fourth pharyngeal pouches in the early stages of embryonic development, which can cause underdevelopment or absence of the thymus and parathyroid glands. The thymus produces T-cell lymphocytes and decreased thymic volume is associated with lymphopenia. Immune deficiencies vary in severity but typically involve reduced levels of CD3+, CD4+, and CD8+ T lymphocytes, resulting in mildly impaired cellular immunity 28-30. The correlation between immune status and phenotypic characteristics yielded conflicting results 1,3,9. According to Suksawat et al. 31, hypocalcemia patients exhibited higher odds ratios for CD4 lymphopenia. Our study found no correlation between immune status and heart disease, palate anomalies, hormonal function tests, or hypocalcemia. However, we observed that all patients noted to have thymic hypoplasia had low absolute counts or percentages of T cells. In the present study, 66.7% of patients exhibited low absolute lymphocyte counts, while 37.0% had decreased absolute counts or percentages of T cells. A previous study reported decreased CD4+ T cells in 61% of patients, with 52% and 45% exhibiting reductions in CD3+ and CD8+ T cells, respectively 30. Given the high frequency of T cell lymphopenia observed in 22q11.2DS in both our study and previous reports, we recommend immunologic evaluation including enumeration of lymphocyte subsets by flow cytometry for all patients with this syndrome 1,3.

Some 22q11.2DS patients may experience transiently low IgG levels or selective IgM or IgA deficiency 28,29. Our study found that 59.3% of patients had decreased immunoglobulin levels, consistent with previous reports 29-30. However, a prior investigation in Bangkok by Suksawat et al. 31 observed abnormal immunoglobulin levels in just 14% of individuals with 22q11.2DS, which normalized by 1.4 years of age. Additionally, B cell abnormalities have been frequently documented in other studies of 22q11.2DS 32. As patients with 22q11.2DS get older, their immune system tends to improve, and most of them eventually have normal levels of functional T lymphocytes 9,31,33. Nevertheless, the clinical course may be unpredictable, and T-cell counts may not reliably predict the likelihood of infection 9, 34.

Less than 1% of 22q11.2DS patients have a severe T-cell deficiency, which can lead to severe combined immunodeficiency disease due to congenital athymia 28. This condition requires thymic transplant/implant for treatment 28. Early diagnosis is crucial in ensuring the implementation of appropriate management and improving patient outcomes. According to previous studies, a frequent manifestation of the condition is recurring infections, primarily affecting the sinopulmonary system, such as recurring pneumonia and otitis media 3. Our study found no cases of severe infections in individuals with 22q11.2DS. Instead, most only experienced relatively minor infections.

Although live vaccines are typically well tolerated in patients with 22q11.2DS, they should be avoided in those with severe T cell deficiencies 1,3. The current study population exhibited no adverse reactions to live vaccines, likely due to the absence of patients with severely low T cell counts (minimum CD3, CD4, and CD8 levels observed were 607, 395, and 219/cu.mm, respectively). However, the sample size was small and may have precluded inclusion of the approximately 0.5% of 22q11.2DS patients with profound T cell lymphopenia, as estimated previously 28.

Previous studies have demonstrated that individuals with 22q11.2DS can exhibit various intellectual abilities, ranging from borderline intelligence to mild learning disabilities 33,35. Reports suggest that individuals with 22q11.2DS frequently experience cognitive impairment, learning difficulties, intellectual disabilities, speech, and language deficits, as well as mood, behavioral, and psychiatric disorders 1,3,9. The findings of this study confirmed the previously observed decrease in FSIQ 35-37. Furthermore, there was a notable discrepancy between VIQ and FSIQ scores, with the 7- to 17-year-old group having a higher FSIQ than VIQ and the >17-year-old group having a higher VIQ score than FSIQ scores. Our findings revealed no significant correlation between cardiovascular disease and intellectual status in the studied patient population. Interestingly, patients above the age of 17 years old had a lower median FSIQ than those in the younger age group, possibly indicating underdiagnosis or previous inadequate interventions. However, these results should be interpreted with caution due to the small sample size. Each of our patients had a documented history of delayed global development, with 80% presenting with intellectual disabilities (FSIQ, <70).

Congenital heart disease has been shown to impact cognitive function and neurological development. However, our analysis revealed no statistically significant difference in intellectual status between patients with major and minor cardiovascular diseases. Although one adult patient in our study had a history of psychiatric disorders, the sample size was too small to draw statistically significant conclusions regarding psychiatric comorbidities in 22q11.2DS. Nonetheless, as more data on adult populations become available, the incidence of such comorbidities is increasingly being recognized 9. One in every four adults with 22q11.2DS develops schizophrenia 38,39.

Recent clinical practice guidelines on the immunological management of 22q11.2DS recommend an initial evaluation of T, B, and natural killer (NK) cell counts along with naïve and memory T cell subsets 40. Follow-up assessments at periodic intervals are advised to monitor changes in immune function over time. Patients with 22q11.2DS face an increased risk of humoral immune deficiencies later in childhood and adulthood, highlighting the importance of ongoing immune surveillance 40. Administration of live attenuated vaccines including measles-mumps-rubella (MMR) and varicella is endorsed when T cell numbers satisfy minimum criteria, as these vaccines confer protection against wild-type infections 40. A minority of patients with 22q11.2DS have congenital athymia and may benefit from thymic transplantation, a procedure shown to be effective for immune reconstitution and survival in this population 40.

Because this was a cross-sectional study, there were significant limitations due to the lack of longitudinal data on the patient's immunologic, hormonal, and intellectual evaluation. As the 22q11.2DS progresses throughout the lifespan, new syndrome-related conditions may emerge, immunologic and hormonal conditions may improve with age, and psychological problems may appear later in adult life 3,9. There was a selection bias because only patients with a suspected clinical phenotype underwent FISH evaluation, and most of the sample was obtained from a pediatric cardiology clinic. As a result, determining the actual prevalence of 22q11.2DS and discerning clinical differences between patients with detected and undetected 22q11.2 deletions cannot be statistically validated. Further prospective longitudinal studies with more diverse groups are necessary to obtain more precise data on patients with 22q11.2DS in our region.

## Conclusion

Our study in Taiwan has demonstrated a diverse range of clinical manifestations of 22q11.2DS. Therefore, in any individual presenting with suspected associated symptoms, such as cardiac defects, abnormal facial features, thymic hypoplasia, cleft palate, or hypocalcemia, genetic testing for 22q11.2DS should be considered to confirm the diagnosis and allow for appropriate genetic counseling. Early diagnosis and intervention are crucial in minimizing morbidity and improving the quality of life for affected individuals. Given the multisystem nature of 22q11.2DS, we recommend that patients be evaluated regularly by a multidisciplinary care team for comprehensive management.

## Figures and Tables

**Table 1:** Historical and clinical profiles (*n* = 27)

Clinical characteristics	*n* (%)
Gender: male	20 (74.1)
Median age (yr [IQR])	17.9 (14.1-20.3)
Median age of diagnosis (yr [IQR])	0.8 (0.3-7.2)
Parental history of 22q11.2 DS	
Confirmed 22q11 deletion	2 (7.4)
Facial malformation	2 (7.4)
Facial dysmorphic features	
Typical facial malformation	25 (92.6)
Cleft lip	1 (3.7)
Cleft palate	7 (25.9)
Cleft lip and palate	1 (3.7)
History of cardiac surgery	11 (40.7)
Presence of thymus (*n* = 11)	
Normal	7 (63.6)
Absence or ectopic	4 (36.4)
History of recurrent infection	11 (40.7)
Recurrent pneumonia	3 (11.1)
Acute otitis media	5 (18.5)
Chronic otitis media	1 (3.7)
Septicemia	1 (3.7)
History of vaccine adverse effects	1 (3.7)

**Table 2:** Cardiovascular observations (*n* = 27)

The type of cardiovascular anomalies	N (%)	Associated lesions: n (%)						
		RAA	Double aortic arch	ASCA	Bilateral SVC	LSVC	MAPCAs	Subpulmonic VSD
Congenital heart disease	20 (74.1)							
Tetralogy of Fallot and variants								
TOF/PS	5 (18.5)	1 (3.7)	1 (3.7)	1 (3.7)	1 (3.7)	—	—	2 (7.4)
TOF/PA	6 (22.2)	1 (3.7)	—	—	—	1 (3.7)	1 (3.7)	—
Interrupted aortic arch type B	1 (3.7)	—	—	—	—	—	1 (3.7)	—
Ventricular septal defect	11 (40.7)	2 (7.4)	1 (3.7)	1 (3.7)	1 (3.7)	—	1 (3.7)	2 (7.4)
Atrial septal defect	4 (14.8)	1 (3.7)	1 (3.7)	1 (3.7)	-	1 (3.7)	1 (3.7)	2 (7.4)
Patent ductus arteriosus	3 (11.1)	—	—	—	—	—	—	1 (3.7)
Vascular anomalies								
Aberrant subclavian artery	1 (3.7)	1 (3.7)	—	—	—	—	—	—
No congenital heart disease	7 (25.9)							

*Note*. ASCA, aberrant subclavian artery; LSVC, left superior vena cava; MAPCAs, major aortopulmonary collateral arteries; PA, pulmonary atresia; PS, pulmonary stenosis; RAA, right-sided aortic arch; SVC, superior vena cava; TOF, tetralogy of Fallot; VSD, ventricular septal defect.

**Table 3:** Immunological and laboratory characteristics (*n* = 27)

Parameters	*N* (%)
Abnormal thyroid function	
Hypothyroidism	1 (3.7)
Hyperthyroidism	3 (11.1)
Hypoparathyroidism	4 (14.8)
Hypocalcemia	11 (40.7)
Immunologic parameters	
Low white blood cell count	0 (0.0)
Low absolute lymphocyte count	18 (66.7)
T-cell deficiency	
Low CD3	3 (11.1)
Low CD4	3 (11.1)
Low CD8	4 (14.8)
Low immunoglobulin	
Low IgG	4 (14.8)
Low IgA	5 (18.5)
Low IgM	4 (14.8)
Low IgE	3 (11.1)

**Table 4:** Cognitive function (*n* = 6)

Intellectual status	*N* (%)
Median age of evaluation (yr [IQR])	5.6 (3.4-8.1)
Median full-scale IQ/DQ level (IQR)	67 (62-74)
-1 to -2 *SD*	3 (50.0)
Less than -2 *SD*	1 (16.7)
